# Asymmetric Dimethylarginine (ADMA) as a Novel Risk Factor for Progression of Coronary Artery Calcification in Patients with Chronic Kidney Disease

**DOI:** 10.3390/jcm14041051

**Published:** 2025-02-07

**Authors:** Shuzo Kobayashi, Takayasu Ohtake, Yasuhiro Mochida, Kunihiro Ishioka, Machiko Oka, Kyoko Maesato, Hidekazu Moriya, Sumi Hidaka

**Affiliations:** Department of Kidney Disease & Transplant Center, Shonan Kamakura General Hospital, Kamakura 247-8533, Japan; shuzo@shonankamakura.or.jp (S.K.); yasuhiro.mochida@tokushukai.jp (Y.M.); kunihiro.ishioka@tokushukai.jp (K.I.); m_oka@shonankamakura.or.jp (M.O.); zwr10460@nifty.ne.jp (K.M.); h_moriya@shonankamakura.or.jp (H.M.); s_hidaka@shonankamakura.or.jp (S.H.)

**Keywords:** chronic kidney disease, coronary artery calcification, asymmetric dimethylarginine, risk factor, progression

## Abstract

**Background:** Vascular calcification (VC) is a characteristic feature of atherosclerosis in patients with chronic kidney disease (CKD), and coronary artery calcification (CAC) significantly impacts future cardiovascular events and mortality. Although factors associated with CAC are well reported, only a few studies have evaluated the factors associated with the progression of CAC in pre-dialysis patients with CKD. **Methods:** We quantitatively evaluated CAC progression using the CAC score (CACS) measured using 16-row multi-detector computed tomography and assessed associated factors in 74 patients with CKD. **Results:** The median annual increase in CACS was 23.7 (IQR 2.0–73.0). CAC progression was associated with serum phosphate and plasma asymmetric dimethylarginine (ADMA) levels, an endogenous inhibitor of nitric-oxide synthase and a marker of endothelial dysfunction and atherosclerosis, in univariate analysis. Multivariate analysis revealed that ADMA is an independent risk factor for CAC progression in patients with CKD. The annual change in CACS was significantly different between patients with ADMA values <0.51 and those with ADMA values >0.51 (*p* < 0.05). Elevated ADMA levels were also significantly associated with estimated glomerular filtration rate (eGFR) decline in the univariate analysis. **Conclusions:** ADMA is a novel risk factor for CAC progression in patients with CKD. Vascular endothelial cell dysfunction, represented by elevated ADMA levels, may contribute to the progression of vascular calcification in patients with pre-dialysis CKD.

## 1. Introduction

Coronary artery calcification (CAC) is a marker of severity of atherosclerotic vascular disease and is a predictor of future adverse cardiovascular events in patients undergoing dialysis [1,2]. However, CAC has also been observed in patients with chronic kidney disease (CKD) at an early stage, prior to the initiation of renal replacement therapy [3,4,5]. In predialysis patients with CKD, CAC significantly impacts the risk of future cardiovascular disease (CVD), myocardial infarction, heart failure, and all-cause mortality [6,7,8].

The factors associated with CAC in patients with pre-dialysis CKD have not been fully elucidated. In a previous study, we reported that the CAC score (CACS) in patients with CKD was markedly elevated when the glomerular filtration rate (GFR) decreased below 60 mL/min/1.73 m^2^, and a significant inverse relationship existed between CACS and GFR in a cross-sectional observational study [9]. CAC becomes more prevalent and severe with a decline in GFR. Furthermore, we found that plasma asymmetric dimethylarginine (ADMA), an endogenous inhibitor of nitric-oxide synthase [10], and insulin resistance, represented by the homeostasis model assessment-insulin resistance (HOMA-IR) [10], were significantly correlated with CAC in pre-dialysis patients with CKD, along with serum phosphate, calcium × phosphate, plasma fibrinogen, and GFR [9]. Plasma ADMA levels were negatively correlated with GFR and positively correlated with CACS [9]. In a cross-sectional study, multivariate regression analysis determined HOMA-IR to be an independent contributing factor to CACS in patients with pre-dialysis CKD patients [9].

Although CAC has a strong impact on the prognosis of patients with CKD, only a few reports have evaluated the risk factors for CAC progression in pre-dialysis CKD patients [8,11,12]. Therefore, this study aimed to evaluate the factors associated with CAC progression in patients with non-dialysis-dependent CKD. Characteristic feature of CAC in patients with CKD is medial calcification associated with osteoblastic transformation of vascular smooth muscle cells, and the role of endothelium on CAC has not been elucidated in patients with CKD. In our previous cross-sectional study, ADMA showed a significant association with CAC in pre-dialysis CKD patients [9]. However, we aimed to further elucidate the role of endothelial dysfunction on CAC by evaluating the associating factors for CAC progression in patients with CKD. We hypothesized that the endothelial dysfunction, represented as elevated ADMA levels, might closely associate with the pathogenesis of CAC progression in patients with CKD. Herein, we suggest that the plasma level of ADMA is a novel risk factor for CAC progression in pre-dialysis patients with CKD.

## 2. Materials and Methods

### 2.1. Patients Enrollment and Purpose of This Study

**Figure 1:**
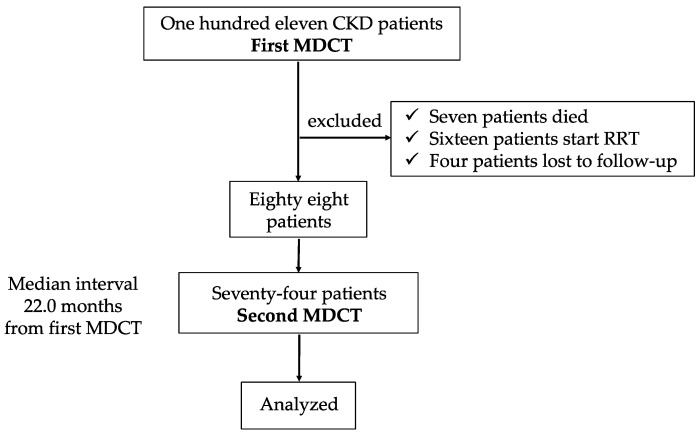
Study protocol. Seventy-four patients were included in this study. CACS was evaluated twice with a median interval of 22.0 months (IQR 20.0–25.0).

This was a single-center, retrospective cohort study. Eligible patients included 111 individuals with CKD patients who underwent a 16-row multidetector computed tomography (MDCT) to evaluate CACS between March 2006 and June 2006 at our hospital. During the follow-up period, 3 patients died, 16 patients underwent renal replacement therapy (12 patients on hemodialysis and 4 patients on peritoneal dialysis), and 4 patients were lost to follow up. Among resting 88 patients, 74 underwent a second MDCT scan with a median interval of 22.0 months, and all these patients were enrolled in the study (Figure 1). The study conformed to the principles of the Declaration of Helsinki and was approved by the institutional review board (IRB) of our medical group (TGE02630-024). The IRB allowed an opt-out approach to obtain consent from patients, and patients could opt out after the research project was posted on our hospital’s website.

### 2.2. Measurement of Coronary Artery Calcification

MDCT was performed twice: at baseline and during follow-up after a median interval of 22.0 months (IQR 20.0–25.0). Patients with stent implantation and/or arrhythmia, including atrial fibrillation or uncontrollable tachycardia, were excluded from the CACS evaluation using MDCT. MDCT was performed using with a LightSpeed Ultrafast 16 (General Electric Medical System, Tokyo, Japan), and the Agatston score was calculated [13] following the method described by Horiguchi et al. [14], as in our previous study [9]. Briefly, volumetric data for the entire heart were obtained in helical mode with the following parameters: 1.25-mm collimation width × 16 detectors, gantry rotation speed of 0.5 s per rotation, 120 kV, and 100 mA. The pitch was varied according to the heart rate. Slices, 2.5 mm in thickness, were reconstructed at 2.5 mm intervals, with the center of the temporal window corresponding to 80% of the R-R interval. The calcium score, volume, and mass were determined from the MDCT data on a commercially available workstation (Adventure Windows, version 4.4.1; General Electric Medical System, Tokyo, Japan) using CAC scoring software (version 3.5; Smartscore, Tokyo, Japan). To measure the area and peak density of the plaques, the regions of interest were set by vessels and slices, with a threshold for pixels greater than 130 Hounsfield units, according to the Agatston method. The CACS was expressed as the sum of the calcification scores of the four epicardial coronary arteries.

### 2.3. Patient Assessment and Laboratory Data

Information, including age, sex, cause of CKD, comorbidities, and medications, was obtained from medical records. Physiological data, including body weight, height, and blood pressure (systolic and diastolic), were recorded. Laboratory parameters, including serum creatinine (sCr), estimated glomerular filtration rate (eGFR), serum albumin, calcium (Ca), phosphate (Pi), intact parathyroid hormone (iPTH), ADMA, high-sensitivity C-reactive protein (hsCRP), and HOMA-IR were measured at the time of the first MDCT after overnight fasting for at least 12 h. EDTA-treated plasma samples were used to measure glucose, insulin, lipids, and ADMA levels, while other parameters were measured using serum samples. Insulin levels were measured by radioimmunoassay (RIA) (Insulin RIA-BEAD 2; Dinabot, Tokyo, Japan), and hsCRP was measured using a nephelometric immunoassay. Concentrations of ADMA in plasma were measured by HPLC by pre-column derivatization with o-phthalaldehyde, after the removal of plasma samples with carboxylic acid solid-phase extraction cartridges. The assay was performed outside the laboratory (SRL, Tokyo, Japan). The detection limit of this assay was 0.1 µmol/L. HOMA-IR was calculated using the following formula: HOMA-IR = fasting glucose (mmol/L) × fasting insulin (µU/mL)/22.5 [15]. eGFR was calculated by the following formula: 194 × Cr^−0.194^ × age^−0.287^ in men, and 194 × Cr^−0.194^ × age^−0.287^ × 0.739 in women [16].

### 2.4. Statistical Analysis

Continuous data were expressed as mean ± standard deviation (SD) or median (interquartile range: IQR) as appropriate, and categorical variables are presented as numbers (percentage). Skewed variables or variables with wide SD including hsCRP, HOMA-IR, and iPTH were logarithmic transformation before analysis. Comparison of continuous variables was performed using Wilcoxon rank-sum tests. Correlation between CACS progression (delta CACS) and variables were analyzed using univariate regression analysis. Serum phosphate and ADMA were significant associating factors, and diabetes, eGFR, and serum calcium showed borderline association with CAC progression in univariate analysis. These factors may potentially confound each other because diabetes exacerbates endothelial dysfunction (represented as ADMA), eGFR decline associates with elevation of ADMA and serum phosphate, and diabetes may promote eGFR decline. Therefore, multivariate regression analysis was performed to control these potential confounders and determine the independent associating factors on the CAC progression. Parameters with *p* value < 0.1 in univariate analysis including diabetes, eGFR, serum calcium, serum phosphate, and ADMA were used as independent variables. Data analysis was performed using SPSS ver. 22.0 (SPSS Inc., Chicago, IL, USA), and a *p* value < 0.05 was considered statistically significant.

## 3. Results

### 3.1. Baseline Characteristics of Enrolled Patients

The baseline characteristics are shown in Table 1. The study included 74 patients (46 men, 28 women) with a mean age of 67.0 ± 11.0 years old. Median levels of sCr and eGFR were 2.02 mg/dL and 38.0 mL/min/1.73 m^2^, respectively. Twenty-seven patients (36.5%) had diabetes mellitus, and 58 patients (79.4%) had hypertension. The mean serum Ca and Pi levels were within the normal ranges, whereas the median iPTH (81.0 pg/mL) was slightly higher than the normal range (10–65 pg/mL). The median HOMA-IR (2.22) was higher than the normal range (<1.6), and there were 30 patients (40.5%) with insulin resistance (defined as HOMA-IR > 2.5). Median ADMA was 0.51 with IQR 0.44–0.57. The CACS at baseline showed a skewed distribution with a median value of 42.7 with IQR 0–274.2 at first MDCT. Twenty-one patients (28.4%) had no CACS, and the maximum CACS value was 2563.2 at first MDCT.

### 3.2. Association Between CAC Progression and Parameters

The second CACS and the annual change in CACS were 101.4 (2.6–356.3) and 23.7 (2.0–73.0), respectively (Table 2). Among 21 patients with 0 CACS at baseline, 14 patients (66.7%) showed 0 CACS at the second MDCT, and the remaining 7 patients showed positive CACS at the second MDCT. Although the change in eGFR from the first to second MDCT was not different (*p* = 0.27) between the group with zero CACS at the first and second MDCT (14 patients) and the group with CACS increased at the second MDCT from 0 CACS at the first MDCT (remaining 7 patients), ADMA values at baseline showed a borderline association (*p* = 0.059) with the increase in CACS from 0 at the first MDCT to positive scores at the second MDCT (Table 3).

Univariate analysis using baseline parameters at first MDCT revealed that serum phosphate and ADMA levels were significantly associated with CACS progression (Table 4). Diabetes, eGFR, and serum Ca levels tended to correlate with the progression of CACS (*p* < 0.1), although the correlations were not statistically significant. Multivariate regression analysis, including parameters with *p* values < 0.1 as variables, revealed that ADMA was an independent predictive factor of CACS progression (*p* = 0.048) (Table 4).

### 3.3. CAC Progression and Kidney Function Decline According to Stratified ADMA

**Figure 2:**
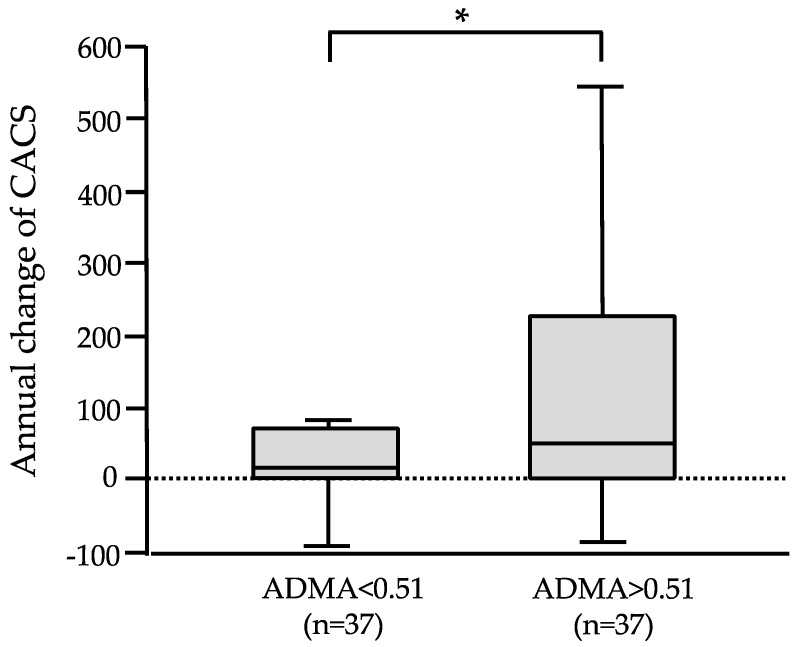
Stratified association between ADMA and CACS progression. CAC progression was significantly different according to the stratified ADMA value. Higher ADMA was significantly associated with higher annual change in CACS compared with those in lower ADMA. * *p* < 0.05.

Annual changes in CACS were evaluated according to the stratification by median ADMA values (higher ADMA group and lower ADMA group) (Figure 2). The progression of CACS differed significantly between patients with ADMA < 0.51 [13.3 (0–63.4)] and those with ADMA > 0.51 [47.1 (0–223.6)] (*p* < 0.05).

### 3.4. Associating Factors for eGFR Decline

From the baseline median eGFR value 38.0 mL/min/1.73 m^2^ (IQR 26.5–52.2), the median eGFR changed to 27.9 mL/min/1.73 m^2^ (IQR 15.7–42.0) at the second MDCT in the study subjects. As a result, the annual change in eGFR from the first MDCT to second MDCT was −6.23 mL/min/1.73 m^2^ (IQR −14.2–2.3) (Table 2). We evaluated the factors associated with the decline in eGFR. Univariate regression analysis indicated that diabetes mellitus (r = 0.357, *p* = 0.003), diastolic blood pressure (r = −0.26, *p* = 0.035), serum Ca (r = −0.268, *p* = 0.028), serum Pi (r = 0.245, *p* = 0.045), HOMA-IR (r = 0.299, *p* = 0.015), and ADMA (r = 0.332, *p* = 0.012) were significantly associated with eGFR decline. eGFR decline was not associated with the baseline CACS. However, eGFR decline showed a borderline association with an annual change in CACS (r = 0.64, *p* = 0.061) (Table 5). The multivariate regression analysis indicated that diastolic blood pressure was an independent predicting factor of eGFR decline (β = −0.611, *p* = 0.021). Although not statistically significant, ADMA showed a borderline association with eGFR decline in the multivariate regression analysis (β = 49.0, *p* = 0.076).

## 4. Discussion

CAC is an important risk factor for future cardiovascular events, including cardiac infarction and heart failure, which significantly affect the prognosis of patients with pre-dialysis CKD [6,7,8]. Therefore, factors associated with CAC in this population should be accurately and precisely evaluated. However, only a few studies have evaluated the risk factors for CAC progression in patients with pre-dialysis CKD [8,11,12]. In our study, CAC progression was significantly associated with serum phosphate and ADMA levels in the univariate analysis, and multivariate analysis revealed that ADMA was an independent risk factor for CAC progression in patients with pre-dialysis CKD. Elevated plasma concentrations of ADMA are known markers of endothelial dysfunction and atherosclerosis, and are associated with overall mortality and cardiovascular events in hemodialysis patients [17]. Our findings suggest that ADMA is a novel independent risk factor for CAC progression in patients with pre-dialysis CKD. ADMA might not only be related to vascular endothelial dysfunction but also to vascular calcification, which concomitantly leads to characteristic features of atherosclerosis in patients with CKD, a condition often referred to as vascular failure [18].

The underlying mechanisms of ADMA in vascular calcification have not yet been fully elucidated. However, limited observational studies have indicated a relationship between elevated ADMA levels and vascular calcification [9,19,20,21]. Iribarren et al. evaluated the relationship between CAC and ADMA in young adults entering middle age [19], finding that ADMA levels were significantly higher in patients with CAC than in those without CAC. Cohen et al. [20] and Kumagai et al. [21] examined the relationship between ADMA and vascular calcification in patients undergoing hemodialysis. ADMA was significantly correlated with CAC [20] and aortic calcification [21] in patients undergoing hemodialysis. ADMA was also significantly and positively correlated with PTH [20]. Vascular calcification, along with diabetic vascular lesions, represent major complications in patients with CKD, leading to the development of a high prevalence of skin ulcers in this population [22]. In our study, diabetes showed a borderline association with CAC progression in patients with CKD. Diabetes exacerbates endothelial dysfunction and promotes vascular calcification [23]. In this respect, endothelial dysfunction (elevation of ADMA levels) caused by CKD and/or diabetes may have a strong impact on the progression of CAC in patients.

To date, no other studies have evaluated the relationship between CAC and ADMA levels in patients with pre-dialysis CKD. In our study, CAC and its progression were significantly correlated with plasma ADMA levels. Recent studies indicated an association between endothelial dysfunction and vascular calcification [24,25]. Bergh et al. have reported that endothelial dysfunction aggravates arterial media calcification in warfarin-treated rats [26]. A warfarin-supplemented diet induced arterial media calcification in rats, and the simultaneous administration of L-NAME and warfarin significantly aggravated media calcification. The authors concluded that early changes in endothelial function significantly affect the progression of arterial media calcification. Yuan et al. reported that endothelial cells participate in vascular calcification through endothelial-mesenchymal transition, cytokine secretion, extracellular vesicle synthesis, angiogenesis regulation and hemodynamics [27]. The key factor in vascular calcification is the transformation of vascular smooth muscle cells (VSMC) into osteoblast-and chondrocyte-like cells. In this context, cross-talk between vascular endothelial cells and VSMC may occur to maintain their integrity. ADMA is typically used as a surrogate marker for endothelial dysfunction. Therefore, endothelial dysfunction, represented by elevated ADMA levels induced by CKD, may promote VSMC transformation into osteoblast-like cells. Therefore, future studies should verify the pathogenesis of vascular calcification in patients with CKD.

The risk factors for CAC progression in pre-dialysis patients with CKD in previous reports include diabetes mellitus, decline in GFR, fibroblast growth factor (FGF) 23, interleukin (IL)-6, tumor necrosis factor (TNF)-alpha reported by Bundy et al. [11], diabetes mellitus reported by Kestenbaum et al. [12], and serum phosphate reported by Russo et al. [8]. In our study, serum phosphate levels were significantly associated with CAC progression, and diabetes and eGFR showed borderline significance. Inflammation, represented by hsCRP, is not associated with CAC progression. Bundy et al. reported that a decline in kidney function and inflammation may be independently associated with CAC progression, apart from existing atherosclerotic risk factors [11]. The difference between the report by Bundy et al. and ours might be caused by the sample size of the study. Our study included 74 patients, while the report by Bundy et al. included 1123 patients in participants from the CRIC study. In patients undergoing hemodialysis, inflammation is a strong risk factor for CAC progression [26]. The inflammatory status caused by the uremic milieu or oxidant stress in pre-dialysis CKD patients might be mild compared with that in advanced stages of CKD, such as in patients undergoing hemodialysis. As one of the mechanisms of increased inflammation in patients with moderate to advanced CKD, a recent study by Obeid et al. provided the potential role of gut bacteria-mediated metabolites on inflammation and cardiovascular morbidity in patients with CKD [27].

In our study, we could not determine whether VC predicted eGFR decline. CAC and CAC progression did not predict the eGFR decline in our study. The eGFR decline was correlated with diabetes, diastolic blood pressure, serum calcium, serum phosphate, HOMA-IR, and ADMA in the univariate analysis. Park et al. recently reported the relationship between CAC progression and kidney function decline [28]. They evaluated 1173 patients with CKD stages G1–G5, and they found that the incidence of kidney failure with renal replacement therapy was significantly higher in patients with CAC progression. Therefore, CAC progression may be associated with an increased risk of CKD development.

This study had some limitations. First, this was a single-center retrospective study with a small sample size. This may limit the generalizability of the findings, and the detection of factors associated with CAC progression compared to other larger-scale studies. Second, retrospective design may have introduced bias, as patients with more severe disease may have been overrepresented. Third, we did not evaluate inflammatory parameters such as IL-6 and TNF-alpha. Therefore, the association with inflammation was assessed solely based on hsCRP levels in our study. Fourth, because we could not evaluate the ADMA values at the time of second MDCT, we could not determine whether ADMA values changed during the study period and could not elucidate how the change in ADMA values contributed to the progression of CAC. Finally, the CACS on MDCT could not discriminate between intimal and medial calcifications. Despite these limitations, our findings provide new insights into atherosclerosis progression in patients with CKD. In the future, larger scale, more diverse cohorts are necessary to confirm the findings obtained in this study.

## 5. Conclusions

CAC progression is significantly associated with elevated ADMA levels. ADMA is a novel risk factor for CAC progression in patients with CKD.

## Figures and Tables

**Table 1:** Baseline characteristics.

Variables		(n = 74)
Age (years old)		67.0 ± 11.0
Sex (male:female)		46:28
Comorbidity, n (%)		
	Hypertension	58 (79.4)
	Diabetes	27 (36.5)
Smoking		7 (9.6)
BMI		23.0 ± 3.1
Blood pressure (mmHg)		
	Systolic	131.0 ± 12.0
	Diastolic	75.0 ± 8.0
Laboratory parameters		
	Creatinine (mg/dL)	2.02 (1.47–2.67)
	eGFR (mL/min/1.73 m^2^)	38.0 (26.5–52.2)
	Albumin (g/dL)	4.1 ± 0.4
	T.cho (mg/dL)	203.0 (172.0–227.0)
	TG (mg/L)	127.0 (97.0–171.0)
	HDL-C (mg/dL)	52.3 (41.9–63.9)
	LDL-C (mg/dL)	115.0 (89.0–137.0)
	Ca (mg/dL)	9.2 ± 0.5
	Pi (mg/dL)	3.6 ± 0.7
	iPTH (pg/mL)	81.0 (58.0–146.0)
	Fibrinogen (mg/dL)	337.0 (283.0–384.0)
	hsCRP (mg/dL)	0.08 (0.033–0.174)
	urinary protein (g/gCr)	0.39 (0.20–1.28)
	ADMA (µmol/L)	0.51 (0.44–0.57)
	HOMA-IR	2.22 (1.10–5.90)
Medication		
	ARB	62 (85.0)
	Statin	26 (36.0)
CACS		42.7 (0–274.2)

Data are expressed as n (%), mean ± SD, or median (IQR). Abbreviations: BMI, body mass index; Ca, calcium; Pi, phosphate; eGFR, estimated glomerular filtration rate; iPTH, intact parathyroid hormone; hsCRP, high-sensitive C-reactive protein; ADMA, asymmetric dimethylarginine; HOMA-IR, homeostasis model assessment-insulin resistance; CACS, coronary artery calcification score.

**Table 2:** Changes of CACS and kidney function.

	First MDCT	Second MDCT	Annual Change
CACS	42.7 (0–274.2)	101.4 (2.6–356.3)	23.7 (2.0–73.0)
Cr (mg/dL)	2.02 (1.47–2.67)	2.32 (1.64–3.47)	0.09 (−0.006–0.59)
eGFR (mL/min/1.73 m^2^)	38.0 (26.5–52.2)	27.9 (15.7–42.0)	−6.23 (−14.2–2.3)

Data are expressed as median (IQR). Abbreviations: CACS, coronary artery calcification score; Cr, creatinine; eGFR, estimated glomerular filtration rate.

**Table 3:** Change in eGFR and ADMA in patients with zero CACS at first MDCT.

	CACS Zero to Zero Score			CACS Zero to Positive Score			
	(n = 14)			(n = 7)			
	at first MDCT	at second MDCT	delta change	at first MDCT	at second MDCT	delta change	*p*
eGFR(mL/min/1.73 m^2^)	47.9 ± 29.8	31.6 ± 23.8	−16.3 ± 36.7	29.9 ± 13.3	28.4 ± 11.6	−1.5 ± 9.2	0.26
ADMA(µmol/L)	0.47 ± 0.05			0.53 ± 0.07			0.059

**Table 4:** Associating factors of CACS progression.

	Univariate Regression Analysis		Multivariate Regression Analysis	
	r	*p*	β	*p*
Age	0.129	0.33		
Diabetes	0.228	0.08	1.38	0.17
Smoking	0.02	0.85		
Systolic BP	0.109	0.41		
Diastolic BP	−0.181	0.17		
Cr	0.129	0.33		
eGFR	−0.224	0.08	0.048	0.78
Albumin	−0.062	0.64		
T.cho	−0.199	0.13		
TG	0.01	0.94		
HDL-C	−0.175	0.18		
LDL-C	−0.192	0.14		
Ca	−0.211	0.09	−0.033	0.84
Pi	0.264	0.04 *	0.174	0.25
Ca×Pi	0.093	0.48		
iPTH	0.006	0.97		
hsCRP	−0.021	0.87		
Fibrinogen	0.024	0.86		
HOMA-IR	−0.083	0.53		
ADMA	0.389	0.005 *	0.303	0.048 *
ARB	0.145	0.27		
Statin	0.003	0.98		

Abbreviations: BP, blood pressure; Cr, creatinine; eGFR, estimated glomerular filtration ratio; T.cho, total cholesterol; TG, triglyceride; HDL-C, high-density lipoprotein; LDL-C, low-density lipoprotein; Ca, calcium; Pi, phosphate; iPTH, intact parathyroid hormone; hsCRP, high-sensitive C-reactive protein; HOMA-IR, homeostasis model assessment-insulin resistance; ADMA, asymmetric dimethylarginine; ARB, angiotensin receptor blocker; * *p* < 0.05.

**Table 5:** Associating factors of eGFR decline.

(n = 74)	Univariate Regression Analysis	
Baseline Parameters	R	*p*
Age	0.109	0.38
Diabetes	0.356	0.003 *
Systolic BP	0.014	0.91
Diastolic BP	−0.26	0.035 *
Albumin	−0.057	0.65
Ca	−0.268	0.028 *
Pi	0.245	0.045 *
hsCRP	−0.008	0.95
HOMA-IR	0.299	0.015 *
ADMA	0.332	0.012 *
Baseline CACS	0.079	0.53
annual change in CACS	0.64	0.061
ARB	0.02	0.87
Urinary protein	0.104	0.4

Abbreviations; BP, blood pressure; Ca, calcium; Pi, phosphate; hsCRP, high-sensitive C-reactive protein; HOMA-IR, homeostasis model assessment insulin resistance; ADMA, asymmetric dimethyl arginine; CACS, coronary artery calcification score; ARB, angiotensin receptor blocker. * *p* < 0.05.

## Data Availability

All data can be supplied for reasonable requests.

## References

[1] Goodman G.W., Goldin J., Kuizon D.B., Yoon C., Gales B., Sider D., Wang Y., Chung J., Emerick A., Greaser L. (2000). Coronary artery calcification in young adults with end-stage renal disease who are undergoing dialysis. N. Engl. J. Med..

[2] Raggi P., Boulay A., Chasan-Taber S., Amin N., Dillon M., Burke S.K., Chertow G.M. (2002). Cardiac calcification in adult hemodialysis patients: A link between end-stage renal disease and cardiovascular disease?. J. Am. Coll. Cardiol..

[3] Bursztyn M., Motro M., Grossman E., Shemesh J. (2003). Accelerated coronary artery calcification in mildly reduced renal function of high-risk hypertensives: A 3-year prospective observation. J. Hypertens..

[4] Russo D., Palmiero G., Blasio A.P.D., Balletta M.M., Andreucci V.E. (2004). Coronary artery calcification in patients with CRF not undergoing dialysis. Am. J. Kidney Dis..

[5] Fox C.S., Larson M.G., Keyes M.G., Levy D., Clouse M.E., Culleton B., O’Donnell C.J. (2004). Kidney function is inversely associated with coronary artery calcification in men and women free of cardiovascular disease: The Framingham Study. Kidney Int..

[6] Chen J., Budoff M.J., Reilly M.P., Yang W., Rosas S.E., Rahman M., Zhang X., Roy J.A., Lustigova E., Nessel L. (2017). Coronary artery calcification and risk of cardiovascular disease and death among patients with chronic kidney disease. JAMA Cardiol..

[7] Tian L., Jaeger B.C., Scialla J.J., Budoff M.J., Mehta R.C., Jaar B.G., Saab G., Dobre M.A., Reilly M.P., Rader D.J. (2024). CRIC Study Investigators: Progression of coronary artery calcification and risk of clinical events in CRIC Renal Insufficiency Cohort Study. Am. J. Kidney Dis..

[8] Russo D., Corrao S., Miranda I., Ruocco C., Manzi S., Elefante R., Brancaccio D., Cozzolino M., Biondi M.L., Andreucci V.E. (2007). Progression of coronary artery calcification in predialysis patients. Am. J. Nephrol..

[9] Kobayashi S., Oka M., Maesato K., Ikee R., Mano T., Moriya H., Ohtake T. (2008). Coronary artery calcification, ADMA, and insulin resistance in CKD patients. Clin. J. Am. Soc. Nephrol..

[10] Stuhlinger M.C., Abbasi M.C., Chu J.W., Lamendola C., Mclaughlin T.L., Cooke J.P., Reaven G.M., Tsao P.S. (2002). Relationship between insulin resistance and an endogenous nitric oxide synthase inhibitor. JAMA.

[11] Bundy J.D., Chen J., Yang W., Budoff M., Go A.S., Grunwald J.E., Kallem R.R., Post W.S., Reilly M.P., Ricardo A.C. (2018). Risk factors for progression of coronary artery calcification in patients with chronic kidney disease: The CRIC study. Atherosclerosis.

[12] Kestenbaum B.R., Adeney K.L., de Boer L.H., Ix J.H., Shlipak M.G., Siscovick D.S. (2009). Incidence and progression of coronary calcification in chronic kidney disease: The Multi-Ethnic Study of Atherosclerosis. Kidney Int..

[13] Agatston A.S., Janowitz W.R., Hildner F.J., Zusmer N.R., Viamonte M., Detrano R. (1990). Quantification of coronary artery calcium using ultrafast computed tomography. J. Am. Coll. Cardiol..

[14] Horiguchi J., Yamamoto H., Akiyama Y., Marukawa K., Hirai N., Ito K. (2004). Coronary artery calcium scoring using 16-MDCT and a retrospective ECG-gating reconstruction algorithm. Am. J. Roentgenol..

[15] Matthews D.R., Hosker J.P., Rudenski A.S., Naylor B.A., Treacher D.F., Turner R.C. (1985). Homeostasis model assessment: Insulin resistance and beta-cell function from fasting plasma glucose and insulin concentration in man. Diabetologia.

[16] Matsuo S., Imai E., Horio M., Yasuda Y., Tomita K., Nitta K., Yamagata K., Tomino Y., Yokoyama H., Hishida A. (2009). Revised equations for estimated GFR from serum creatinine in Japan. Am. J. Kidney Dis..

[17] Zoccali C., Bode-Böger S.M., Mallamaci F., Benedetto A., Tripepi G., Malatino L.S., Cataliotti A., Bellanuova I., Fermo I., Frölich J.C. (2001). Plasma concentration of asymmetrical dimethylarginine and mortality in patients with end-stage renal disease: A prospective study. Lancet.

[18] Kobayashi S. (2016). Cardiovascular events in chronic kidney disease (CKD)—An importance of vascular calcification and microcirculatory impairment. Ren. Replace. Ther..

[19] Iribarren C., Husson G., Sydow K., Wang B.Y., Sidney S., Cooke J.P. (2007). Asymmetric dimethyl-arginine and coronary artery calcification in young adults entering middle age: The CARDIA Stury. Eur. J. Cardiovasc. Prev. Rehabil..

[20] Coen G., Mantella D., Sardella D., Beraldi M.P., Ferrari I., Pierantozzi A., Lippi B., Giulio S.D. (2009). Asymmetric dimethylarginine, vascular calcifications and parathyroid hormone serum levels in hemodialysis patients. J. Nephrol..

[21] Kumagai H., Sakurai M., Takita T., Maruyama Y., Uno S., Ikegaya N., Hishida A. (2006). Association of homocysteine and asymmetric dimethylarginine with atherosclerosis and cardiovascular events in maintenance hemodialysis patients. Am. J. Kidney Dis..

[22] Mancin S., Mazzoleni B., Reggiani F., Calatroni M., Alterchi E., Donizzetti D., Finazzi S., Soekeland F., Sguanci M., Badalamanti S. (2023). Integrated protocol for the prevention and treatment of skin ulcers in patients with end-stage renal disease. MethodsX.

[23] Yahagi K., Kolodgie F.D., Lutter C., Mori H., Romero M., Finn A.V., Virmani R. (2017). Pathology of human coronary and carotid artery atherosclerosis and vascular calcification in diabetes mellitus. Arter. Thromb. Vasc. Biol..

[24] Bergh G.V.D., Branden A.V.D., Opdebeeck B., Fransen P., Neven E., De Meyer G.R.Y., D’Haese P.C., Verhulst A. (2022). Endothelial dysfunction aggravates arterial media calcification in warfarin administered rats. FASEB J..

[25] Yuan C., Ni L., Zhang C., Hu X., Wu X. (2021). Vascular calcification: New insight into endothelial cells. Microvasc. Res..

[26] Ohtake T., Ishioka K., Honda K., Oka M., Maesato K., Mano T., Ikee R., Moriya H., Hidaka S., Kobayashi S. (2010). Impact of coronary artery calcification in hemodialysis patients: Risk factors and associations with prognosis. Hemodial. Int..

[27] Obeid R., Awwad H., Heine G.H., Emrich I.E., Fliser D., Zawada A.M., Geisel J. (2024). Plasma concentrations of Trimethylamine-N-oxide, choline, and betaine in patients with moderate to advanced chronic kidney disease and their relation to cardiovascular and renal outcomes. J. Ren. Nutr..

[28] Park C.H., Kim H.W., Park J.T., Chang T.I., Yoo T.H., Park S.K., Lee K.B., Jung J.Y., Jeong J.C., Oh K.H. (2024). KNOW-CKD Investigators: Association between progression of coronary artery calcification and development of kidney failure with replacement therapy: Findings from KNOW-CKD study. Atherosclerosis.

